# Where is it and How Does it Get There – Intracellular Localization and Traffic of P-glycoprotein

**DOI:** 10.3389/fonc.2013.00321

**Published:** 2013-12-30

**Authors:** Dong Fu

**Affiliations:** ^1^Faculty of Pharmacy, The University of Sydney, Sydney, NSW, Australia

**Keywords:** P-glycoprotein, intracellular localization, traffic, recycling, cell polarization, multidrug resistance in cancer

## Abstract

P-glycoprotein (P-gp), an ATP-binding cassette, is able to transport structurally and chemically unrelated substrates. Over-expression of P-gp in cancer cells significantly decreases the intercellular amount of anticancer drugs, and results in multidrug resistance in cancer cells, a major obstacle in cancer chemotherapy. P-gp is mainly localized on the plasma membrane and functions as a drug efflux pump; however, P-gp is also localized in many intracellular compartments, such as endoplasmic reticulum, Golgi, endosomes, and lysosomes. P-gp moves between the intracellular compartments and the plasma membrane in a microtubule-actin dependent manner. This review highlights our current understanding of (1) the intracellular localization of P-gp; (2) the traffic and cycling pathways among the cellular compartments as well as between these compartments and the plasma membrane; and (3) the cellular factors regulating P-gp traffic and cycling. This review also presents a potential implication in overcoming P-gp-mediated multidrug resistance by targeting P-gp traffic and cycling pathways and impairing P-gp localization on the plasma membrane.

## Introduction

P-glycoprotein (P-gp), a 170 kDa membrane protein, is a member of sub-family B of the ATP-binding cassette (ABC) transporter superfamily, and is also called ABCB1. P-gp has two structurally identical halves. Its N-terminal half contains six transmembrane domains, followed by a large cytoplasmic domain with an ATP-binding site. Similarly, the C-terminal half also has six transmembrane domains and an ATP-binding site (1, 2). Plasma membrane located P-gp is able to transport many chemically and structurally unrelated substrates out of the cells, and acts as an efflux pump (1, 2). P-gp is primarily expressed in the liver, kidney, gastrointestinal tract, and blood brain barrier. P-gp is located on the canalicular apical membrane of hepatocytes in the liver; on the brush border of proximal tubule cells in the kidney; and on the apical membrane of mucosal cells in the small intestine (3). Given the transporting function of P-gp, these tissue distributions allow P-gp to excrete endogenous metabolites, exogenous substrates, and toxins into the urine, bile, and feces. Thus, P-gp can protect the organism as well as eliminate cellular wastes (3, 4). Furthermore, another essential localization of P-gp is on the luminal surface of capillary endothelial cells of the blood brain barrier which prevents cytotoxins from penetrating the endothelium and protects brain (5).

Although animal well-being, normal physiological function, and life span were not affected after P-gp was knocked out in mice, higher drug sensitivity and increased drug side effect/toxicity occurred (6). While knocking out P-gp appears to be less problematic, over-expression of P-gp causes major concerns in clinical oncology. The most notable consequence of over-expression of P-gp in clinic is to cause multiple drug resistance (MDR) in cancer chemotherapy (2). Given P-gp has a structurally broad range of substrates, the occurrence of MDR during chemotherapy is one of the big challenges for successful cancer treatment in clinic. MDR can be either intrinsic, occurring in cancers that have not been exposed to chemotherapy before but derived from tissue naturally expressing P-gp (e.g., liver, kidney, intestinal cancers), or required MDR, which develops after cancers are treated with chemotherapy (7). Nearly half of human cancers express P-gp at levels sufficient to develop MDR. The likelihood of failure in chemotherapy is increased when P-gp expression is upregulated during therapy (8).

## Intracellular Localization of P-gp

**Figure 1:**
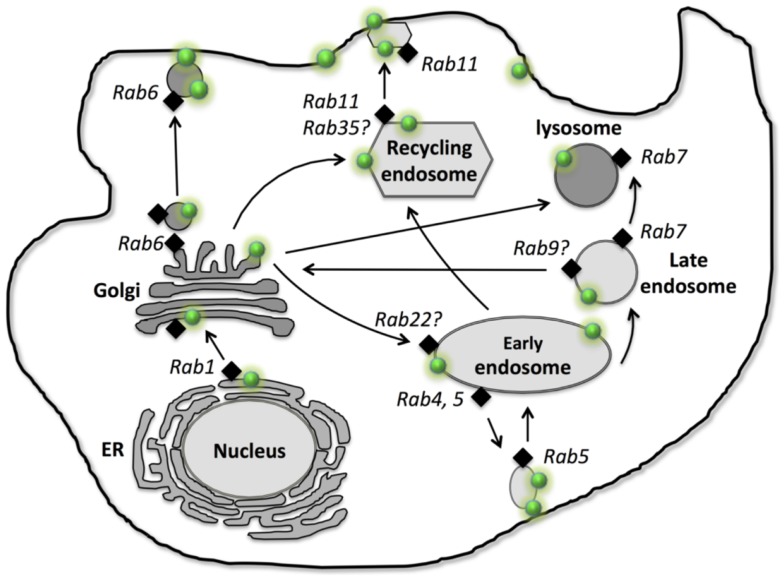
**Intracellular localization, traffic, and recycling of P-glycoprotein**. P-gp is shown as “green dot 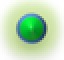.” Different Rab GTPases that are involved in P-gp traffic and recycling are indicated as “black diamond 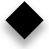.” Arrows represent the traffic and recycling path among the intracellular organelles and between the cellular organelles and the plasma membrane.

P-glycoprotein is primarily localized on the plasma membrane for its efflux function, however, it is also localized intracellularly (9, 10). Using immunofluorescence and over-expression of P-gp-GFP fusion protein approaches, co-localization results revealed that P-gp is localized in many cellular organelles, including endoplasmic reticulum (ER) (9), Golgi (9), early endosome (11–13), recycling endosome (12), later endosome, lysosome (9, 11), and proteasome (14) (Figure 1). These intracellular localizations link to synthesis (ER), modification (Golgi), traffic/recycling (Golgi and endosomes), and degradation (lysosome and proteasome) sites for P-gp. Although one study suggests P-gp is also located in mitochondria in doxorubicin-resistant K562 human leukemia cells (15), others reveal that P-gp is not presented in mitochondria either in MCF-7 (ADR) human breast cancer and KB-V1 human cervix carcinoma drug resistant cell lines (16) or in primary rat hepatocytes (17). Furthermore, transient transfection of P-gp-GFP in cancer cells reveals that the ER and Golgi localization of P-gp appears to be transient, suggesting that P-gp can rapidly traffic to the endosomal compartment and the plasma membrane localization after it is synthesized in ER and modified in Golgi. This rapid transport to the membrane localization explains why less ER or Golgi localization can be observed in the stable cell line which is overexpressed with P-gp-GFP. It is possible that activity of P-gp synthesis remains at a relatively low level due to the very long half-life of P-gp (14–17 h) in the stable cell line (18). Similarly, the degradation localization (lysosome) also appears to be less common within the cells compared to the endosomal localization which is involved in constantly trafficking/recycling P-gp between the cellular pool and the plasma membrane (11).

## Intracellular Traffic and Recycling of P-gp

After synthesis in ER, P-gp first needs to be correctly folded before exit from ER and entry to Golgi for modification. Currently, very little information is available about the exact regulatory process for P-gp folding in ER. Glycoprotein glucosyl transferase (UGGT) is able to sense the folding states of glycoproteins, resulting in mis-folded glycoprotein rebinding calnexin (a chaperon for protein folding) and going through re-folding cycles or being rapidly degraded via endoplasmic-reticulum-associated protein degradation (ERAD) (19). Thus, UGGT may play a potential role in recognizing the folding of P-gp. Moreover, a study suggests that SPTLC1 (Serine palmitoyltransferase enzyme 1) is able to interact with ABCA1 and cause ER retention of ABCA1, revealing the role of SPTLC1 in ER exit of ABC transporters (20). Furthermore, the formation of disulfide bonds is a critical step in the maturation of the majority of the proteins inside ER (21). Studies showed that two other ABC transporters, ABCB6 and ABCC8, form the disulfide bonds between highly conserved cysteine, which is important for these ABC transporters to exit ER and traffic to the plasma membrane (22). We still lack direct evidence of how P-gp export at the ER – the first step of its trafficking along the biosynthetic secretory pathway. In mammalian cells, the ER export occurs via Coat Protein II (COPII)-coated vesicles. COPII vesicles bud from the ER and are able to fuse to ER-Golgi intermediated compartment (23). Studies reveal that COPII plays an essential role in exporting ABCB1 and ABCC7 (cystic fibrosis transmembrane conductance regulator, CFTR) from ER to Golgi (24, 25), suggesting COPII may regulate ER export of P-gp as well. Golgi is involved in the biosynthesis of glycan chains of glycoproteins, the 150 kDa P-gp is transported to the Golgi and glycosylated as the 170 kDa mature protein (26) (Figure 1).

After its modification in Golgi, the 170 kDa mature P-gp traffics to the plasma membrane. Membrane proteins can traffic to the plasma membrane via either the constitutive pathway which involves membrane protein-containing vesicles moving directly to the plasma membrane (27, 28) or the endosomal pathway in which protein-containing vesicles are first transported to endosomal compartments to establish the intracellular pool, and then traffic to the plasma membrane (29). In both cases, the cytoskeleton is needed for the traffic of these membrane protein vesicles (30). The *trans*-Golgi network (TGN) is a major sorting site for proteins trafficking to the plasma membrane and endosomal pathway. A study suggests that membrane proteins use *N*-glycan chains as sorting determinants (31); whether this also applies to P-gp needs to be investigated. However, some studies show that N-glycosylation at amino acid residue asparagine 596 at third extracellular loop is not necessary for ABCG2 traffic (32), and the immature core-glycosylated CFTR (ABCC7) can be transported to the plasma membrane and is functional (33).

In the non-polarized cancer cells, P-gp was reported localized in EEA1 and Rab5 positive early endosome which serves as an intracellular reservoir prior to P-gp moving to the plasma membrane (11), suggesting that P-gp can traffic to the plasma membrane via the indirect endosomal pathway. Furthermore, immunofluorescence study showed that P-gp is also localized in lysosome after transient expression of human P-gp-GFP in HeLa cells or in the human breast cancer MCF-7 cells, which are stably expressed with P-gp-GFP, suggesting P-gp can be moved to the lysosomal degradation compartment, presumably through the early and late endosome (12). However, an immunofluorescence study reveals that P-gp is not localized in Rab11 positive recycling endosomes in human breast cancer MCF-7 cells, which stably express P-gp-GFP (11). In the polarized WIFB9 cells as well as hepatocytes, apical ABC transporters (e.g., P-gp, ABCB11) move to the apical membrane via Rab11a recycling endosomes and recycle between the apical membrane and the Rab11a positive intracellular endosomal pool (29, 34) (Figure 1). These studies suggest that P-gp traffics and recycles via different endosomal pathways (early endosome vs. recycling endosome) in non-polarized cells (e.g., cancer cells) and polarized cells.

## Regulation of P-gp Traffic and Recycling – Role of Rab GTPases

Rab GTPases, the largest branch of small GTPase, are known to regulate vesicular transport in exocytosis, endocytosis, and recycling by controlling many steps in membrane trafficking such as vesicle formation, movement, uncoating, docking, and fusion (35). So far more than 70 Rab GTPases have been identified in humans (35). Each Rab protein is believed to be specifically associated with a particular organelle or pathway (35). Currently, few studies revealed the role of Rab proteins in P-gp trafficking, thus, the more general involvement of Rab proteins in ABC transporters trafficking and recycling will be discussed.

Rab1, 2, and 6 are localized in ER and Golgi and regulate vesicle transport along the ER-Golgi biosynthetic pathway. Rab1, 2 regulate vesicle movement from ER to Golgi (35, 36), while Rab6 is involved in Golgi to the cell surface for exocytosis of newly synthesized proteins and lipids (37). Study showed that P-gp was predominantly intracellular, largely in Rab6-containing Golgi vesicles and Golgi cisternae (7), suggesting Rab6 may regulate P-gp traffic directly from Golgi to the plasma membrane (Figure 1).

Along the endosomal trafficking pathway, Rab11 and Rab13 are involved in membrane protein traffic from Golgi to the recycling endosome (38–40). Rab11a was shown to regulate P-gp and ABCB11 traffic to the apical membrane in polarized WIFB9 cells (29, 34) (Figure 1). Furthermore, studies showed that Rab11a was also needed for both WT-CFTR and ΔF508-CFTR to undergo trafficking to the apical recycling compartment in polarized human airway epithelia cells (41) as well as polarized intestinal epithelial cells (42). These studies reveal that, in polarized cells, P-gp traffics to the apical membrane via the Rab11a positive recycling endosome. However, in non-polarized MCF-7 cells, stable expressed P-gp-EGFP did not co-localize with Rab11 positive recycling endosome (11), suggesting P-gp does not traffic to the cell surface via the Rab11a positive recycling endosome. Evidence indicates that membrane trafficking of CFTR is cell type-specific and it differs in polarized human airway epithelial cells such as CFBE41o- cells and in non-polarized fibroblasts such as BHK-21 cells (41). Thus, Rab11a may have a differential role in P-gp trafficking in non-polarized and polarized cells. Other Rab proteins, such as Rab17, Rab25, Rab35, and Rab40 are also localized in recycling endosome (36, 43). Rab35 is shown to play important role in insulin-stimulated GLUT4 (Glucose transporter type 4) translocation in adipocytes (44). Further studies are needed to investigate the role of Rab11 as well as other Rab proteins (e.g., Rab17, Rab25, Rab35, and Rab40) in P-gp traffic in both polarized and non-polarized cells.

Early endosomal pathway is shown to be involved in P-gp trafficking and recycling. Among many of the early endosomal Rab proteins, Rab4 and Rab5 are known to regulate P-gp trafficking and recycling in many cancer cells (12, 13, 45). Over-expression of dominant-negative Rab5 mutant (S34N-Rab5) results in large intracellular accumulation of P-gp-EGFP in non-polarized Hela cells, and similar cellular accumulation of wild type P-gp in multidrug resistant MCF-7/Adr cells, revealing that Rab5 regulates P-gp exocytosis from the endosome compartment (such as early endosome) to the plasma membrane in non-polarized cells (12). In contrast, another study in colon cancer cells LS174T, demonstrated that over-expression of wild-type Rab5 resulted in recycling P-gp from the plasma membrane into intracellular compartments, suggesting that Rab5 regulates P-gp endocytosis instead of exocytosis (13), however, this study did not investigate the polarization status of the cell culture of LS174T, which is shown to be polarized in normal culture condition (46). Thus, the differential role of Rab5 in P-gp trafficking and recycling in these cancer cells may be related to the difference in polarization (Figure 1).

Rab4 is also localized in early endosome and shown to regulate P-gp exocytosis in drug resistant leukemia cells, K562ADR (45). Cell surface expression of P-gp decreased after over-expression of GFP-Rab4 or constitutively active Rab4Q72L mutant, but not dominant-negative Rab4S27N mutant or Rab14 in the K562ADR cells, suggesting that Rab4 regulates P-gp trafficking to the plasma membrane from endosomal compartments (45). However, in HeLa cells, the intracellular localization of P-gp-EGFP was not affected when there was over-expression of either wild-type Rab4 or dominant-negative mutant N121I-Rab4 respectively (12), indicating Rab4 does not effect P-gp trafficking. The different cell lines used in these studies may be the reason for obtaining different results. Given many other Rab proteins, such as Rab10, 14, 15, 17, 22, and 23 are localized on early endosome (43), it is likely these Rab proteins can also regulate the trafficking and recycling of P-gp between the early endosomal compartment and the plasma membrane in a cell and tissue type-specific manner.

Transmembrane proteins can be transported from late endosome to lysosome which is responsible for degradation of the membrane proteins (47). P-gp is localized in Lamp-2 positive lysosome, revealing its lysosomal dependent degradation pathway (11). Rab7 is localized on late endosome and lysosome, and is essential for later endocytic membrane trafficking from late endosome to lysosome (48, 49), while Rab9 is localized on later endosome and responsible for transit from later endosome to the TGN (50). Although very little is known about the role of Rab7 and 9 on later endocytic membrane trafficking of P-gp, Rab7 and 9 are shown to regulate other ABC transporter trafficking (e.g., CFTR). Using over-expression of wild type and mutant Rab GTPases, a study revealed that CFTR could enter Rab7-dependent late endosomal traffic or Rab9-mediated translocation to the TGN (51), suggesting the role of both Rab7 and 9 in later endocytic trafficking of CFTR. However, the potential roles of Rab7 and 9 on P-gp trafficking need to be investigated (Figure 1).

## Clinical Implications

P-glycoprotein plays an important role in drug excretion and is one of the main causes for MDR in cancer chemotherapy. Different generations of P-gp inhibitors are developed and enter into preclinical and clinical studies. The first generation of P-gp inhibitors, such as verapamil and cyclosporin A, are active substrates of P-gp. Both verapamil and cyclosporin A cause side effects in patients due to high dose of the drugs are need for their inhibition of P-gp (52, 53). The second generation of P-gp inhibitors include valspodar (PSC 833), dexverapamil, and dofequidar fumarate. However, these second generation inhibitors also inhibit drug metabolism enzymes and other ABC transporters, which results in impaired drug metabolism and elimination (54–56). The third generation of P-gp inhibitors, which are currently undergoing clinical trials, include zosuquidar (LY335979), elacridar (GF120918), CBT-1, and XR9576 (57–60). However, some of the trials are unsuccessful in improving therapeutic efficacy (61). For example, trial was stopped in patient with non-small-cell lung cancer due to chemotherapy-related toxicity after administration of XR9576 (60). Thus, there is an urgent need to develop innovative strategies to overcome P-gp-mediated MDR in cancer chemotherapy.

Given P-gp needs to be transported to the plasma membrane so as to efflux the anticancer drugs out of cells, blocking the trafficking of P-gp to its final destination – the plasma membrane location – can be an innovative approach to overcome MDR and improve therapy. There are multiple potential targets along the P-gp traffic pathway. Study revealed that inhibition of P-gp maturation resulted in accumulation of P-gp in Golgi, and this immature P-gp in Golgi was inactive and presumably led to degradation. Consequently there was an increased cellular accumulation of P-gp substrate (62). Experiments also reveal that blocking P-gp traffic to the plasma membrane by interrupting the cytoskeleton highway or modulating Rab activation can cause increased intracellular accumulation of P-gp, resulting in more intracellular retention of anticancer drug (9, 11, 12). Although the intracellular P-gp that is trapped on the way to the plasma membrane remains active, it is likely that intracellular P-gp do not contribute to drug resistance (63). It is essential to identify the regulatory effectors, such as specific Rab GTPases or Rab binding proteins for P-gp traffic and recycling, and to screen potential candidates for targeting these effectors. Therefore, more studies are needed to understand the molecular and cellular mechanisms of P-gp intracellular traffic/cycling and its regulatory factors.

## Conflict of Interest Statement

The author declares that the research was conducted in the absence of any commercial or financial relationships that could be construed as a potential conflict of interest.

## References

[1] Endicott JA Ling V The biochemistry of P-glycoprotein-mediated multidrug resistance. Annu Rev Biochem (1989) 58:137–71 10.1146/annurev.bi.58.070189.001033 2570548

[2] Gottesman MM Pastan I Ambudkar SV P-glycoprotein and multidrug resistance. Curr Opin Genet Dev (1996) 6:610–7 10.1016/S0959-437X(96)80091-8 8939727

[3] Thiebaut F Tsuruo T Hamada H Gottesman MM Pastan I Willingham MC Cellular localization of the multidrug-resistance gene product P-glycoprotein in normal human tissues. Proc Natl Acad Sci U S A (1987) 84:7735–8 10.1073/pnas.84.21.7735 2444983 PMC299375

[4] Schinkel AH Mayer U Wagenaar E Mol CA van Deemter L Smit JJ Normal viability and altered pharmacokinetics in mice lacking mdr1-type (drug-transporting) P-glycoproteins. Proc Natl Acad Sci U S A (1997) 94:4028–33 10.1073/pnas.94.8.4028 9108099 PMC20562

[5] Cordon-Cardo C O’Brien JP Casals D Rittman-Grauer L Biedler JL Melamed MR Multidrug-resistance gene (P-glycoprotein) is expressed by endothelial cells at blood-brain barrier sites. Proc Natl Acad Sci U S A (1989) 86:695–8 10.1073/pnas.86.2.695 2563168 PMC286540

[6] Schinkel AH Smit JJ van Tellingen O Beijnen JH Wagenaar E van Deemter L Disruption of the mouse mdr1a P-glycoprotein gene leads to a deficiency in the blood-brain barrier and to increased sensitivity to drugs. Cell (1994) 77:491–502 10.1016/0092-8674(94)90212-7 7910522

[7] De Rosa MF Sillence D Ackerley C Lingwood C Role of multiple drug resistance protein 1 in neutral but not acidic glycosphingolipid biosynthesis. J Biol Chem (2004) 279:7867–76 10.1074/jbc.M305645200 14662772

[8] Gottesman MM Fojo T Bates SE Multidrug resistance in cancer: role of ATP-dependent transporters. Nat Rev Cancer (2002) 2:48–58 10.1038/nrc706 11902585

[9] Fu D Bebawy M Kable EP Roufogalis BD Dynamic and intracellular trafficking of P-glycoprotein-EGFP fusion protein: implications in multidrug resistance in cancer. Int J Cancer (2004) 109:174–81 10.1002/ijc.11659 14750166

[10] Fu D Arias IM Intracellular trafficking of P-glycoprotein. Int J Biochem Cell Biol (2012) 44:461–4 10.1016/j.biocel.2011.12.009 22212176 PMC3288648

[11] Fu D Roufogalis BD Actin disruption inhibits endosomal traffic of P-glycoprotein-EGFP and resistance to daunorubicin accumulation. Am J Physiol Cell Physiol (2007) 292:C1543–52 10.1152/ajpcell.00068.2006 17122416

[12] Fu D van Dam EM Brymora A Duggin IG Robinson PJ Roufogalis BD The small GTPases Rab5 and RalA regulate intracellular traffic of P- glycoprotein. Biochim Biophys Acta (2007) 1773:1062–72 10.1016/j.bbamcr.2007.03.023 17524504

[13] Kim H Barroso M Samanta R Greenberger L Sztul E Experimentally induced changes in the endocytic traffic of P-glycoprotein alter drug resistance of cancer cells. Am J Physiol (1997) 273:C687–702 9277367 10.1152/ajpcell.1997.273.2.C687

[14] Katayama K Noguchi K Sugimoto Y FBXO15 regulates P-glycoprotein/ABCB1 expression through the ubiquitin – proteasome pathway in cancer cells. Cancer Sci (2013) 104:694–702 10.1111/cas.12145 23465077 PMC7657132

[15] Munteanu E Verdier M Grandjean-Forestier F Stenger C Jayat-Vignoles C Huet S Mitochondrial localization and activity of P-glycoprotein in doxorubicin-resistant K562 cells. Biochem Pharmacol (2006) 71:1162–74 10.1016/j.bcp.2006.01.006 16499877

[16] Paterson JK Gottesman MM P-Glycoprotein is not present in mitochondrial membranes. Exp Cell Res (2007) 313:3100–5 10.1016/j.yexcr.2007.04.019 17512524 PMC2075362

[17] Fu D Mitra K Sengupta P Jarnik M Lippincott-Schwartz J Arias IM Coordinated elevation of mitochondrial oxidative phosphorylation and autophagy help drive hepatocyte polarization. Proc Natl Acad Sci U S A (2013) 110:7288–93 10.1073/pnas.1304285110 23589864 PMC3645550

[18] Muller C Laurent G Ling V P-glycoprotein stability is affected by serum deprivation and high cell density in multidrug-resistant cells. J Cell Physiol (1995) 163:538–44 10.1002/jcp.1041630314 7775597

[19] Dejgaard S Nicolay J Taheri M Thomas DY Bergeron JJ The ER glycoprotein quality control system. Curr Issues Mol Biol (2004) 6:29–42 14632257

[20] Tamehiro N Zhou S Okuhira K Benita Y Brown CE Zhuang DZ SPTLC1 binds ABCA1 to negatively regulate trafficking and cholesterol efflux activity of the transporter. Biochemistry (2008) 47:6138–47 10.1021/bi800182t 18484747 PMC2504083

[21] Wedemeyer WJ Welker E Narayan M Scheraga HA Disulfide bonds and protein folding. Biochemistry (2000) 39:7032 10.1021/bi005111p 10841785

[22] Fukuda Y Aguilar-Bryan L Vaxillaire M Dechaume A Wang Y Dean M Conserved intramolecular disulfide bond is critical to trafficking and fate of ATP-binding cassette (ABC) transporters ABCB6 and sulfonylurea receptor 1 (SUR1)/ABCC8. J Biol Chem (2011) 286:8481–92 10.1074/jbc.M110.174516 21199866 PMC3048732

[23] Budnik A Stephens DJ ER exit sites – localization and control of COPII vesicle formation. FEBS Lett (2009) 583:3796–803 10.1016/j.febslet.2009.10.038 19850039

[24] Tanaka AR Kano F Ueda K Murata M The ABCA1 Q597R mutant undergoes trafficking from the ER upon ER stress. Biochem Biophys Res Commun (2008) 369:1174–8 10.1016/j.bbrc.2008.03.018 18343215

[25] Ameen N Silvis M Bradbury NA Endocytic trafficking of CFTR in health and disease. J Cyst Fibros (2007) 6:1–14 10.1016/j.jcf.2006.09.002 17098482 PMC1964799

[26] Molinari A Cianfriglia M Meschini S Calcabrini A Arancia G P-glycoprotein expression in the Golgi apparatus of multidrug-resistant cells. Int J Cancer (1994) 59:789–95 10.1002/ijc.2910590614 7989120

[27] Kipp H Arias IM Newly synthesized canalicular ABC transporters are directly targeted from the Golgi to the hepatocyte apical domain in rat liver. J Biol Chem (2000) 275:15917–25 10.1074/jbc.M909875199 10748167

[28] Kipp H Pichetshote N Arias IM Transporters on demand: intrahepatic pools of canalicular ATP binding cassette transporters in rat liver. J Biol Chem (2001) 276:7218–24 10.1074/jbc.M007794200 11113123

[29] Sai Y Nies AT Arias IM Bile acid secretion and direct targeting of mdr1-green fluorescent protein from Golgi to the canalicular membrane in polarized WIF-B cells. J Cell Sci (1999) 112(Pt 24):4535–45 10574703 10.1242/jcs.112.24.4535

[30] Musch A Cohen D Rodriguez-Boulan E Myosin II is involved in the production of constitutive transport vesicles from the TGN. J Cell Biol (1997) 138:291–306 10.1083/jcb.138.2.291 9230072 PMC2138203

[31] Keller P Simons K Post-golgi biosynthetic trafficking. J Cell Sci (1997) 110(Pt 24):3001–9 9365270 10.1242/jcs.110.24.3001

[32] Diop NK Hrycyna CA N-linked glycosylation of the human ABC transporter ABCG2 on asparagine 596 is not essential for expression, transport activity, or trafficking to the plasma membrane. Biochemistry (2005) 44:5420–9 10.1021/bi0479858 15807535

[33] Gee HY Noh SH Tang BL Kim KH Lee MG Rescue of DeltaF508-CFTR trafficking via a GRASP-dependent unconventional secretion pathway. Cell (2011) 146:746–60 10.1016/j.cell.2011.07.021 21884936

[34] Wakabayashi Y Dutt P Lippincott-Schwartz J Arias IM Rab11a and myosin Vb are required for bile canalicular formation in WIF-B9 cells. Proc Natl Acad Sci U S A (2005) 102:15087–92 10.1073/pnas.0503702102 16214890 PMC1257697

[35] Hutagalung AH Novick PJ Role of Rab GTPases in membrane traffic and cell physiology. Physiol Rev (2011) 91:119–49 10.1152/physrev.00059.2009 21248164 PMC3710122

[36] Stenmark H Rab GTPases as coordinators of vesicle traffic. Nat Rev Mol Cell Biol (2009) 10:513–25 10.1038/nrm2728 19603039

[37] Grigoriev I Splinter D Keijzer N Wulf PS Demmers J Ohtsuka T Rab6 regulates transport and targeting of exocytotic carriers. Dev Cell (2007) 13:305–14 10.1016/j.devcel.2007.06.010 17681140

[38] Ullrich O Reinsch S Urbe S Zerial M Parton RG Rab11 regulates recycling through the pericentriolar recycling endosome. J Cell Biol (1996) 135:913–24 10.1083/jcb.135.4.913 8922376 PMC2133374

[39] Schonteich E Wilson GM Burden J Hopkins CR Anderson K Goldenring JR The Rip11/Rab11-FIP5 and kinesin II complex regulates endocytic protein recycling. J Cell Sci (2008) 121:3824–33 10.1242/jcs.032441 18957512 PMC4365997

[40] Nokes RL Fields IC Collins RN Folsch H Rab13 regulates membrane trafficking between TGN and recycling endosomes in polarized epithelial cells. J Cell Biol (2008) 182:845–53 10.1083/jcb.200802176 18779367 PMC2528589

[41] Swiatecka-Urban A Brown A Moreau-Marquis S Renuka J Coutermarsh B Barnaby R The short apical membrane half-life of rescued {Delta}F508-cystic fibrosis transmembrane conductance regulator (CFTR) results from accelerated endocytosis of {Delta}F508-CFTR in polarized human airway epithelial cells. J Biol Chem (2005) 280:36762–72 10.1074/jbc.M508944200 16131493

[42] Silvis MR Bertrand CA Ameen N Golin-Bisello F Butterworth MB Frizzell RA Rab11b regulates the apical recycling of the cystic fibrosis transmembrane conductance regulator in polarized intestinal epithelial cells. Mol Biol Cell (2009) 20:2337–50 10.1091/mbc.E08-01-0084 19244346 PMC2669039

[43] Jean S Kiger AA Coordination between RAB GTPase and phosphoinositide regulation and functions. Nat Rev Mol Cell Biol (2012) 13:463–70 10.1038/nrm3379 22722608

[44] Davey JR Humphrey SJ Junutula JR Mishra AK Lambright DG James DE TBC1D13 is a RAB35 specific GAP that plays an important role in GLUT4 trafficking in adipocytes. Traffic (2012) 13:1429–41 10.1111/j.1600-0854.2012.01397.x 22762500 PMC3470861

[45] Ferrandiz-Huertas C Fernandez-Carvajal A Ferrer-Montiel A Rab4 interacts with the human P-glycoprotein and modulates its surface expression in multidrug resistant K562 cells. Int J Cancer (2011) 128:192–205 10.1002/ijc.25310 20209493

[46] Baas AF Kuipers J van der Wel NN Batlle E Koerten HK Peters PJ Complete polarization of single intestinal epithelial cells upon activation of LKB1 by STRAD. Cell (2004) 116:457–66 10.1016/S0092-8674(04)00114-X 15016379

[47] Settembre C Fraldi A Medina DL Ballabio A Signals from the lysosome: a control centre for cellular clearance and energy metabolism. Nat Rev Mol Cell Biol (2013) 14:283–96 10.1038/nrm3565 23609508 PMC4387238

[48] Feng Y Press B Wandinger-Ness A Rab 7: an important regulator of late endocytic membrane traffic. J Cell Biol (1995) 131:1435–52 10.1083/jcb.131.6.1435 8522602 PMC2120682

[49] Bucci C Thomsen P Nicoziani P McCarthy J van Deurs B Rab7: a key to lysosome biogenesis. Mol Biol Cell (2000) 11:467–80 10.1091/mbc.11.2.467 10679007 PMC14786

[50] Barbero P Bittova L Pfeffer SR Visualization of Rab9-mediated vesicle transport from endosomes to the trans-Golgi in living cells. J Cell Biol (2002) 156:511–8 10.1083/jcb.200109030 11827983 PMC2173336

[51] Gentzsch M Chang XB Cui L Wu Y Ozols VV Choudhury A Endocytic trafficking routes of wild type and DeltaF508 cystic fibrosis transmembrane conductance regulator. Mol Biol Cell (2004) 15:2684–96 10.1091/mbc.E04-03-0176 15075371 PMC420093

[52] Tan B Piwnica-Worms D Ratner L Multidrug resistance transporters and modulation. Curr Opin Oncol (2000) 12:450–8 10.1097/00001622-200009000-00011 10975553

[53] Thomas H Coley HM Overcoming multidrug resistance in cancer: an update on the clinical strategy of inhibiting p-glycoprotein. Cancer Control (2003) 10:159–65 12712010 10.1177/107327480301000207

[54] Krishna R Mayer LD Multidrug resistance (MDR) in cancer. Mechanisms, reversal using modulators of MDR and the role of MDR modulators in influencing the pharmacokinetics of anticancer drugs. Eur J Pharm Sci (2000) 11:265–83 10.1016/S0928-0987(00)00114-7 11033070

[55] Bates S Kang M Meadows B Bakke S Choyke P Merino M A Phase I study of infusional vinblastine in combination with the P-glycoprotein antagonist PSC 833 (valspodar). Cancer (2001) 92:1577–90 10.1002/1097-0142(20010915)92:63.0.CO;2-H 11745237

[56] Wandel C Kim RB Kajiji S Guengerich P Wilkinson GR Wood AJ P-glycoprotein and cytochrome P-450 3A inhibition: dissociation of inhibitory potencies. Cancer Res (1999) 59:3944–8 10463589

[57] Morschhauser F Zinzani PL Burgess M Sloots L Bouafia F Dumontet C Phase I/II trial of a P-glycoprotein inhibitor, Zosuquidar.3HCl trihydrochloride (LY335979), given orally in combination with the CHOP regimen in patients with non-Hodgkin’s lymphoma. Leuk Lymphoma (2007) 48:708–15 10.1080/10428190701190169 17454628

[58] Kuppens IE Witteveen EO Jewell RC Radema SA Paul EM Mangum SG A phase I, randomized, open-label, parallel-cohort, dose-finding study of elacridar (GF120918) and oral topotecan in cancer patients. Clin Cancer Res (2007) 13:3276–85 10.1158/1078-0432.CCR-06-2414 17545533

[59] Shukla S Wu CP Ambudkar SV Development of inhibitors of ATP-binding cassette drug transporters: present status and challenges. Expert Opin Drug Metab Toxicol (2008) 4:205–23 10.1517/17425255.4.2.205 18248313

[60] Nobili S Landini I Giglioni B Mini E Pharmacological strategies for overcoming multidrug resistance. Curr Drug Targets (2006) 7:861–79 10.2174/138945006777709593 16842217

[61] Palmeira A Sousa E Vasconcelos MH Pinto MM Three decades of P-gp inhibitors: skimming through several generations and scaffolds. Curr Med Chem (2012) 19:1946–2025 10.2174/092986712800167392 22257057

[62] Loo TW Clarke DM The human multidrug resistance P-glycoprotein is inactive when its maturation is inhibited: potential for a role in cancer chemotherapy. FASEB J (1999) 13:1724–32 10506575 10.1096/fasebj.13.13.1724

[63] Larsen AK Escargueil AE Skladanowski A Resistance mechanisms associated with altered intracellular distribution of anticancer agents. Pharmacol Ther (2000) 85:217–29 10.1016/S0163-7258(99)00073-X 10739876

